# 1393. Loss to Follow-up Rate in the Treatment of Latent Tuberculosis by Region of Origin

**DOI:** 10.1093/ofid/ofab466.1585

**Published:** 2021-12-04

**Authors:** Hikari Yoshii, Charles Bark

**Affiliations:** Case Western Reserve University MetroHealth Medical Center, Lakewood, Ohio

## Abstract

**Background:**

Adherence in the treatment of latent tuberculosis infection (LTBI) is closely related to reactivation and infection control in the population. However, there has been little research on which populations are at higher risk of loss to follow-up. The aim of this study is to investigate how the adherence of LTBI patients in the United States (US) differs by region of origin.

**Methods:**

A retrospective, observational study was conducted from 2001 to 2020. LTBI patients were identified from the Cuyahoga County Tuberculosis Clinic in Cleveland, Ohio. Only patients who were informed of the diagnosis of LTBI were included. Patients were discharged from the Tuberculosis outpatient clinic upon completion of treatment or when the physician decided to discontinue treatment. We defined loss to follow-up as a case where LTBI was diagnosed but the patient was not formally discharged. Patients whose treatment was interrupted due to side effects were not considered loss to follow-up. Odds ratios were calculated using a multivariable regression model with patients from North America as the reference group.

**Results:**

Of 4018 LTBI patients, 1171 (28.7%) were lost to follow-up, of which 950/2314 (41.0%) were from North America. Compared with LTBI patients from North America, significantly lower loss to follow-up rates were observed for those from Middle East and North Africa 30/170 (17.7% OR 0.52, 95% Confidence Interval (CI) 0.31-0.89), South Asia 60/692 (8.7% OR 0.41, 95% CI 0.21-0.78), and Sub-Saharan Africa 69/526 (13.1% OR 0.22, 95% CI 0.14-0.36).

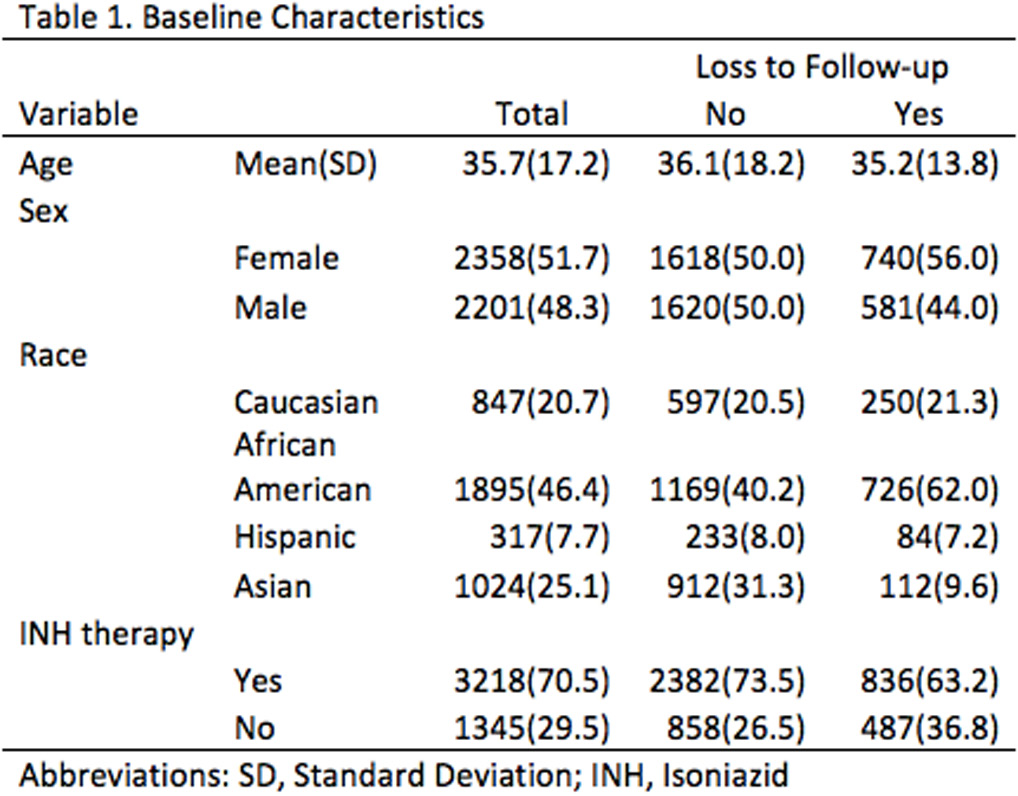

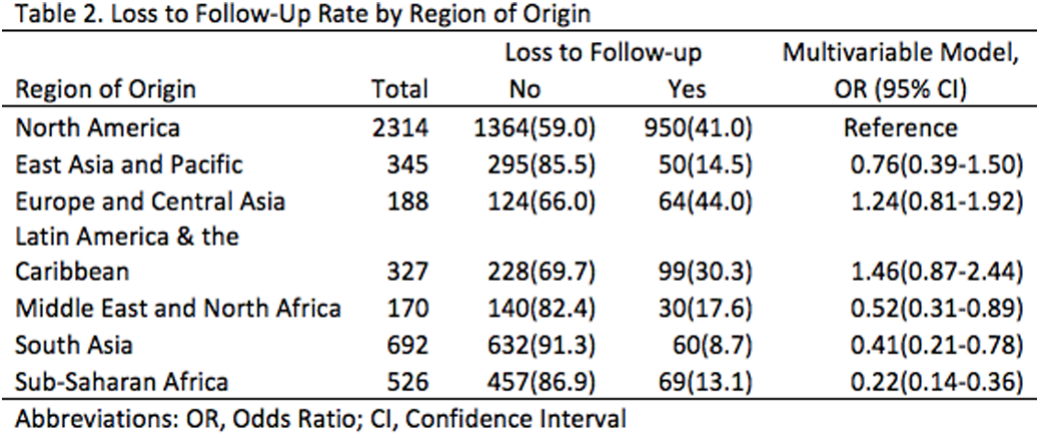

**Conclusion:**

The analysis showed that a high loss to follow-up rate was observed in the patient groups from North America, Europe and Central Asia, and Latin America & the Caribbean. LTBI patients from North America had a significantly higher loss to follow-up rate than those from Middle East and North Africa, South Asia, and Sub-Saharan Africa, respectively. Further research is needed to determine how to intervene in the poorly adherent patient population, such as LTBI patients from North America, Europe and Central Asia, and Latin America & the Caribbean.

**Disclosures:**

**All Authors**: No reported disclosures

